# A randomized trial on the efficacy of split-body versus full-body resistance training in non-resistance trained women

**DOI:** 10.1186/s13102-022-00481-7

**Published:** 2022-05-14

**Authors:** Helene Pedersen, Marius Steiro Fimland, Brad J. Schoenfeld, Vegard Moe Iversen, Kristoffer Toldnes Cumming, Susanne Jensen, Atle Hole Saeterbakken, Vidar Andersen

**Affiliations:** 1 Faculty of Education, Arts and Sports, Western Norway University of Applied Sciences, PB 133, 6851 Sogndal, Norway; 2 Department of Neuromedicine and Movement Science, Faculty of Medicine and Health Sciences, Norwegian University of Science and Technology, Trondheim, Norway; 3 Department of Health Sciences, Lehman College, Bronx, USA; 4 Department of Public Health and Nursing, Faculty of Medicine and Health Sciences, Norwegian University of Science and Technology, Trondheim, Norway; 5 Faculity of Health and Welfare, Østfold University College, Fredrikstad, Norway

**Keywords:** Resistance training, Training frequency, Split-body, Full-body

## Abstract

**Background:**

The aim of this study was to assess the efficacy of a 12-week upper/lower split- versus a full-body resistance training program on maximal strength, muscle mass and explosive characteristics. Fifty resistance untrained women were pair-matched according to baseline strength and randomized to either a full-body (FB) routine that trained all of the major muscle groups in one session twice per week, or a split-body program (SPLIT) that performed 4 weekly sessions (2 upper body and 2 lower body). Both groups performed the same exercises and weekly number of sets and repetitions. Each exercise was performed with three sets and 8–12 repetition maximum (RM) loading. Study outcomes included maximal strength, muscle mass, jump height and maximal power output.

**Results:**

No between-group differences were found in any of the variables. However, both FB and SPLIT increased mean 1-RM from pre- to post-test in the bench press by 25.5% versus 30.0%, lat pulldown by 27.2% versus 26.0% and leg press by 29.2% versus 28.3%, respectively. Moreover, both FB and SPLIT increased jump height by 12.5% versus 12.5%, upper-body power by 20.3% versus 16.7% and muscle mass by 1.9% versus 1.7%, *p* < 0.01, respectively.

**Conclusions:**

This study did not show any benefits for split-body resistance-training program compared to full-body resistance training program on measures of maximal- and explosive muscle strength, and muscle mass.

*Trial registration*: ISRCTN81548172, registered 15. February 2022.

## Background

Whilst there are several well-defined benefits to performing strength training most people do not regularly participate in this exercise modality, often citing lack of time as the reason [1–3]. Designing time-efficient training programs can therefore be valuable to increasing participation and adherence to a strength training program [2]. Many well-trained athletes divide their training into multiple weekly training sessions, particularly during periods of high training load and/or volume [4].

It is theorized that briefer, more frequent weekly training sessions might allow for training with greater training loads compared to less frequent sessions of a longer duration due to maximal energy utilization and reduced fatigue during exercise [4]. Beyond a given threshold, the quality of training begins to degrade as session duration increases [5]. A split-body routine, where different muscle groups are trained on different days, can help support a high volume of work per muscle group while keeping session duration manageable. In contrast, a full-body routine can be performed, where all muscle groups are trained in the same session [5, 6]. Some research shows superior strength improvement and gains in lean body mass in those performing volume-equated programs with higher frequencies and less volume per session, although these findings are inconsistent [5]. It therefore is possible that a higher session frequency would lead to shorter and more intense sessions with less accumulated fatigue, ultimately resulting in more work performed (i.e. a higher training volume). Proper manipulation of load, volume, rest and frequency is essential to improve muscular hypertrophy and muscle strength [7–11]. However, training frequency may be the variable that has gained the least attention and thus requires further investigation [12, 13].

Training frequency is typically defined as either the total number of weekly resistance training sessions, or the number of times a given muscle group is trained per week [7, 12–17]. Previously, several studies have examined the effects of different strength training frequencies on muscular adaptations and muscle strength [1, 3, 4, 6, 9, 12, 18, 19]. The majority of these studies have compared muscle group frequency and not session frequency. Few studies have examined the effects of session frequency on muscular adaptations [4, 6, 18, 19]. Only one of these studies found between-group differences in muscle mass, muscle strength and jump height [6]. Calder et al. [6] reported greater maximal strength increases in the biceps curl (1-RM) in the group performing a split-body routine (four weekly sessions), and a lower 12-RM knee flexion strength and leg muscle mass in the group performing a total-body routine (two weekly sessions). Nevertheless, there are some methodological issues in the abovementioned studies that need to be adressed. For example the majority of these studies had small sample sizes (five to ten subjects per group) [4, 19] and relatively short intervention periods (3–8 weeks) [4, 18, 19].

It has been suggested that women respond differently to resistance training frequency than men [13]. A meta-analysis by Grgic et al. [13] showed that women are more likely to respond more positively to a higher training frequency compared to men. However, these findings were based on relatively few number of studies with several important limitations e.g., low sample size, resistance-trained women not representative of the general population and equated training volume (same number of sets per week). Further, few of the studies comparing different frequencies with equated training volumes have used non-resistance trained individuals as participants [20]. Consequently it would be of interest to compare resistance training programs with different training frequencies among non-resistance trained women to strengthen research-based recommendations for training.

Therefore, the aim of the current study was to examine the effects of performing two weekly total-body sessions versus four weekly split-body sessions, with an equated weekly training frequency per muscle group, on muscular adaptations in untrained women. Based on previous research [6, 13], we hypothesized that the split-body group would be able to (1) train with progressively heavier loads for the same number of repetitions (i.e. performing a higher total volume load), and thereby (2) would show greater increases in maximal strength, muscle mass, upper-body power output and jump height compared to the full-body group.

## Methods

### Experimental approach to the problem

We employed a randomized intervention trial to assess the efficacy of performing either a full-body (FB) resistance-training program twice per week, or a split-body (SPLIT) program four times per week, with training carried out over a 12-week study period. Each muscle group was exercised the same number of times per week (twice) for an equal number of sets and repetitions in both groups. Participants were pair-matched according to their baseline strength levels and then randomly assigned to one of the two experimental groups. The allocation process was generated by a third person, by drawing notes from an envelope. The SPLIT group performed four exercises targeting the lower extremities twice per week and six exercises targeting the upper body twice per week. Alternatively, the FB group performed all ten exercises in the same session, with sessions carried out twice per week. Maximal muscle strength (1-RM in leg press, Lat pulldown and bench press), muscle mass (bioelectrical impedance), jump height (CMJ) and power output in the bench press (at 15 kgs) were tested pre- and post-intervention. The testing was conducted in the lab of Western Norway University College, Campus Sogndal.

### Subjects

The recruitment started 15/06/2016 and ended 20/08/2016. The participants were recruited through direct personal contact or through social media postings. Fifty young, non-resistance trained women volunteered to participate in the study. This number of participants was justified based on the number of participants in previous, similar studies [1, 12]. After accounting for dropouts, 44 participants (SPLIT: n = 19, height = 167.8 ± 6.4 cm; body mass = 70.9 ± 9.6 kg; age 22.5 ± 3.3 years. FB: n = 25, height = 168.3 ± 4.4 cm; body mass = 66.5 ± 8.5 kg; age 22.9 ± 3.1 years) completed the study. The inclusion criteria required that participants had not performed strength training regularly the previous 6 months, were free of injury or pain that could impair performance during training or testing, were over 18 years of age, and had not reached menopause. All participants were informed orally and in writing about the study’s purpose, risks, and benefits, and signed a written informed consent before being allowed to participate. According to national legislation, an ethical approval from the regional ethical committee was deemed unnecessary [“Act on ethics and integrity in research” and “Act on medical and health research” (www.lovdata.no)]. Importantly, the study was conducted in accordance with the ethical guidelines set by the Western Norway University of Applied Sciences review board and was performed in accordance with the Declaration of Helsinki. The data collection and management was evaluated and recommended by the Norwegian Centre for Research Data (NSD, ref 48876).The study was registered retrospectively as a clinical trial (ISRCTN81548172, 15/02/2022).

### Intervention

The intervention lasted from medio August 2016 and until medio December 20/12/2016. An overview of the exercises and the weekly training schedule for the two groups is presented in Table 1.

**Table 1:** Training protocols

Protocol	Day 1	Day 2	Day 3	Day 4
Split-group	Leg press	Lat pulldown	Leg press	Lat pulldown
	Stiff-legged deadlift	Bench press	Stiff-legged deadlift	Bench press
	Leg extension	Cable seated row	Leg extension	Cable seated row
	Hip thrust	Triceps press and Biceps curl in cable (superset)	Hip thrust	Triceps press and Biceps curl in cable (superset)
Full-body group	Leg press	Leg press		
	Stiff-legged deadlift	Stiff-legged deadlift		
	Leg extension	Leg extension		
	Hip thrust	Hip thrust		
	Lat pulldown	Lat pulldown		
	Bench press	Bench press		
	Cable seated row	Cable seated row		
	Triceps press and Biceps curl in cable (superset)	Triceps press and Biceps curl in cable (superset)		

Before each workout, the participants in the FB group performed a 10-min warm-up that consisted of 5 min on either a treadmill, cycle ergometer, or elliptical machine followed by 5 min on a rowing machine. The SPLIT group performed a 5-min warm-up on either a treadmill, cycle ergometer, or elliptical machine on lower extremity training days and a 5-min warm-up on a rowing machine on the upper body training days [18]. In addition, participants performed a warm-up set (8 repetitions at 50% of 1RM) in the first exercise on lower extremity muscles and upper body muscles. Every other session the FB group alternated between starting with the upper- or the lower body exercises.

The training intervention employed a linear periodization model. Throughout the 12-week study period, participants performed three sets per exercise with a progressive increase in load and decrease in the number of repetitions as follows: 12-RM in the first 4 weeks; 10-RM in the next 4 weeks; and 8-RM in the last 4 weeks [11, 21]. The rest interval between sets lasted approximately 1 min [21, 22]. Each session was manually logged by research assistants to document and guide progression of the load. If the subject completed all the repetitions with proper technique, resistance was increased in the next set by 2.5–5 kg.

The SPLIT group and the FB group completed 98% and 97% of the planned sessions, respectively. Of the completed sessions, 97% were supervised by two professional instructors. The instructors controlled for proper technique as well as progression of load. A minimum of 48 h separated each session for the same muscle group. The participants were encouraged to maintain their usual activity throughout the intervention but were prohibited from performing additional strength training.

### Procedures

#### Maximal strength

Upper- and lower-body strength was assessed by 1-RM testing in leg press, bench press and pulldown using machines (Technogym, leg press and lat pulldown) or free-weights (bench press). A familiarization session was conducted before the first experimental test to help ensure proper technique and determine exercise performance characteristics such as grip width, etc., which were in turn standardized throughout all experimental sessions. Before the 1-RM testing (pre- and post) trials, subjects performed a general warm-up consisting of 5 min on treadmill and 5 min on a rowing machine [18], followed by a specific warm-up consisting of three sets with progressive increases in resistance (12 repetitions at 50% of 1-RM, 8 repetitions at 75% of 1-RM and 3 repetitions at 85% of 1-RM). Rest between each warm-up set was approximately 1 min [23], whereas three to 5 min was given between each 1-RM attempt. When a lift was successful, the load was gradually increased by 2.5–5 kg until failure. A valid 1-RM was established by either incompletion of a subsequent attempt, completion of a subsequent attempt with improper technique (see later), or mutual agreement between the participant and test leaders that a higher load could not be lifted. 1-RM values were determined within five attempts.

The 1-RM test in the leg press (Pure Strength–Leg Press, Technogym) was only tested in the concentric phase. The lift started in the bottom position with the knee angle set to approximately 90°. The subject lifted the weight until her legs were fully extended. There was no requirement for tempo, but the concentric movement had to be performed without stopping. The knees had to align with the toes and the buttocks and upper back had to remain in contact with the seat throughout the lift. The intraclass correlation coefficient (ICC) between the familiarization session and the pre-test was 0.87.

For the bench press, 1-RM testing used a standard bench with a 15-k barbell. The subjects laid on the bench with the buttocks and upper back in contact with the bench at all times. Further, the heels had to remain in contact with the floor throughout the lift. The test started with the arms extended. The bar then was lowered in a controlled tempo until it reached the lower part of the sternum. After a brief pause the subjects were instructed to push the bar explosively up to the starting position [14]. ICC between familiarization session and the pre-test was 0.98.

The lat pulldown was performed on an apparatus using two independent handles (Pure Strength–Pulldown, Technogym). Participants sat upright in the seat with arms extended. The seat height was adjusted so that the participants could place their legs comfortably underneath the leg pad. The test started with a concentric phase whereby the subjects pulled the handles down until they were positioned below the chin, as any further pulling would utilize hip extension. ICC between familiarization session and the pre-test was calculated to be 0.93.

#### Muscle mass

Muscle mass was measured via bioelectric impedance (Tanita MC780 multi frequency segmental body composition analyzer). On a separate day from other testing, the participants met in the morning after having abstained from food and drinks for a minimum 10 h, did not shower, and refrained from intense exercised in the previous 24 h. The time of day was approximately the same for both the pre- and post-test. The participants were measured while wearing shorts, a sports bra, and no shoes or socks [24]. Participants’ descriptive information (age, gender and height) was entered into the software. Once body mass was assessed by the scale, the participants stood upright and relaxed, keeping both arms alongside their body, and a full segmental analysis was performed in less than 20 s [25]. The unit provided an estimate of each participant’s muscle mass (in kilograms), which was used for subsequent analysis. For further details of the procedure and reliability, see Verney et al. [25].

#### Jump test

Jump height was evaluated by performing a counter movement jump (CMJ) on a force platform (MuscleLab Force Plate Model 2, Ergotest Technology AS, Langesund, Norge). The jump height was calculated by the impulse using a software program (MuscleLab Software v8.13; Ergotest Technology AS, Langesund, Norway). Upon signal from the test leader, the participants performed a fast, maximal-effort eccentric–concentric movement with hands kept on the hips throughout each jump. Participants were afforded three attempts, but if the third attempt was the best, they continued with additional trials until the jump height decreased. One minute of rest was given between each attempt. The best attempt was used for analysis.

#### Power test

Maximal power output was measured in the concentric phase of the 1-RM in bench press using a 15 kg load (i.e. only the barbell) with a linear encoder (ET-Enc-02, Ergotest Innovation A/S, Porsgrunn, Norway) attached to the bar. The encoder recorded the position, direction, bar displacement and time in the concentric phase in the lift with a resolution of 0.075 cm and sampling frequency of 100 Hz. A software program calculated the maximal mean power output of the lift (MuscleLab Software v8.13, Ergotest Technology AS, Langesund, Norway).

### Statistical analyses

Analysis was conducted according to the intention to treat principle using linear mixed-effects models. This approach uses all available data for all participants at each time point and accounts for missing values [26]. As recommended for balanced RCT`s with similar timing of the repeated measures for all study participants, any group differences at baseline were assumed to be due to chance and baseline levels were pooled over the two study groups [27]. Thus, the following levels were used in the analysis: baseline, FB after 12 weeks, and SPLIT after 12 weeks. The outcome variable was included as the dependent variable and the interaction between time points and group allocation were included as fixed effects. To account for the covariance within-subjects, participants identity was included as random effect in the analysis. Difference in weekly training volume between groups (FB and SPLIT) was assessed with linear mixed-effects models without pooling of group data at baseline. Training volume was set as the dependent variable, time (week 1–12) and groups, as well as their interaction term, were set as fixed effects, and participant identity was used as random effect.

All results are presented as means with 95% confidence intervals (CI), and significance level set to *p* < 0.05. All statistical analyses were conducted in STATA/IC 16.0 for windows (StataCorp LP, USA), and graphs were made in SigmaPlot Version 14.0 for windows (Systat Software, San Jose, CA).

## Results

**Fig. 1:**
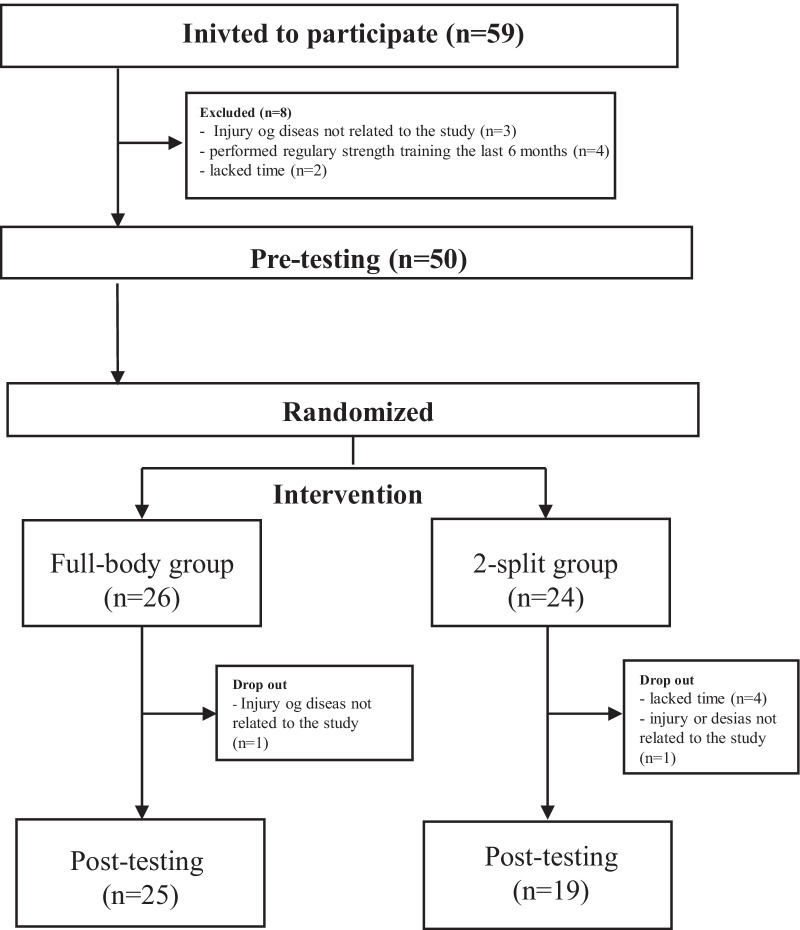
Flowchart showing participants, randomization, and dropouts

The characteristics of the two groups are presented in Table 2. Six subjects did not complete the intervention due to lack of time (four participants), illness or injuries not related to the intervention (two participants) see Fig. 1.

**Table 2:** Baseline characteristics, results are presented as means (SD)

	FB group, n = 26	SPLIT group, n = 24
Age, years	23 (3)	23 (3)
BMI, kg/m^2^	24.0 (3.9)	25.3 (3.8)
Muscle mass, kg	45.9 (4.8)	47.4 (4.6)
Bench press 1RM, kg	36.1 (9.0)	34.5 (7.0)
Leg press 1RM, kg	145.8 (37.9)	153.1 (36.8)
Lat pulldown 1RM, kg	73.7 (12.4)	71.8 (10.1)
Jump performance, cm	20.5 (4.7)	21.1 (5.4)
Power, W	144.4 (31.7)	143.8 (21.4)

*BMI* body mass index, *RM* repetition maximum

### Maximal strength

**Fig. 2:**
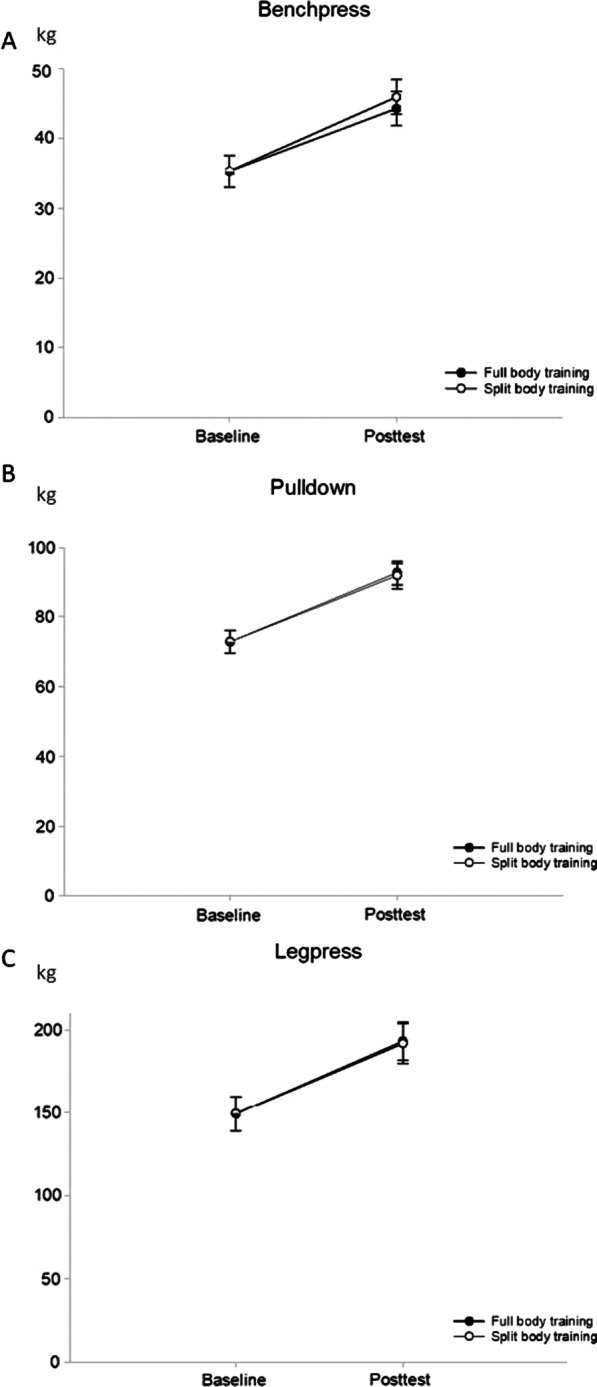
Changes in one repetition maximum strength from baseline to posttest for **A** Bench press, **B** Lat pulldown, and **C** Leg press. Results are presented as mean with 95% CI

Both groups increased their mean 1-RM from pre to post-test in all three exercises (*p* < 0.01), with no statistically significant differences observed between groups (see Fig. 2). Bench press 1-RM increased from 35.3 (33.1, 37.4) kg to 44.4 (41.9, 46.6) kg and 45.9 (43.5, 48.4) kg for FB and SPLIT, respectively. The between-group difference was 1.7 (− 0.1, 3.5) kg, *p* = 0.069. Lat pulldown 1-RM increased from 72.8 (69.6, 76.0) kg to 92.6 (89.0, 96.1) kg and 91.7 (88.0, 95.5) kg for FB and SPLIT, respectively. The between group difference was − 0.8 (− 4.2, 2.5) kg, *p* = 0.629. Leg press 1-RM increased from 149.3 (139.0, 159.6) kg to 193.2 (181.9, 204.5) kg and 191.8 (179.9, 203.8) kg for FB and SPLIT, respectively. The between group difference was − 1.3 (− 11.5, 8.8) kg, *p* = 0.792.

### Jump performance

FB and SPLIT increased mean jump performance from 20.9 (19.3, 22.2) cm to 23.4 (21.8, 52.0) cm and 23.4 (21.7, 25.0) cm, respectively (*p* < 0.01). The mean between group difference was − 0.1 (− 1.5, 1.3) kg, *p* = 0.903.

### Power

FB and SPLIT increased their maximal power output from 144.1 (136.0, 152.2) watt to 173.4 (163.2, 183.6) watt and 168.1 (156.8, 179.5) watt, respectively (*p* < 0.01). The between group difference was − 5.3 (− 8.6, 8.1) watt, *p* = 0.439.

### Muscle mass

FB and SPLIT increased mean total muscle mass from 46.6 (45.3, 47.9) kg to 47.5 (46.2, 48.9) kg and 47.4 (46.1, 48.8) kg, respectively (*p* < 0.01). The mean between group difference was − 0.1 (− 0.8, 0.7) kg, *p* = 0.828.

### Training volume

**Fig. 3:**
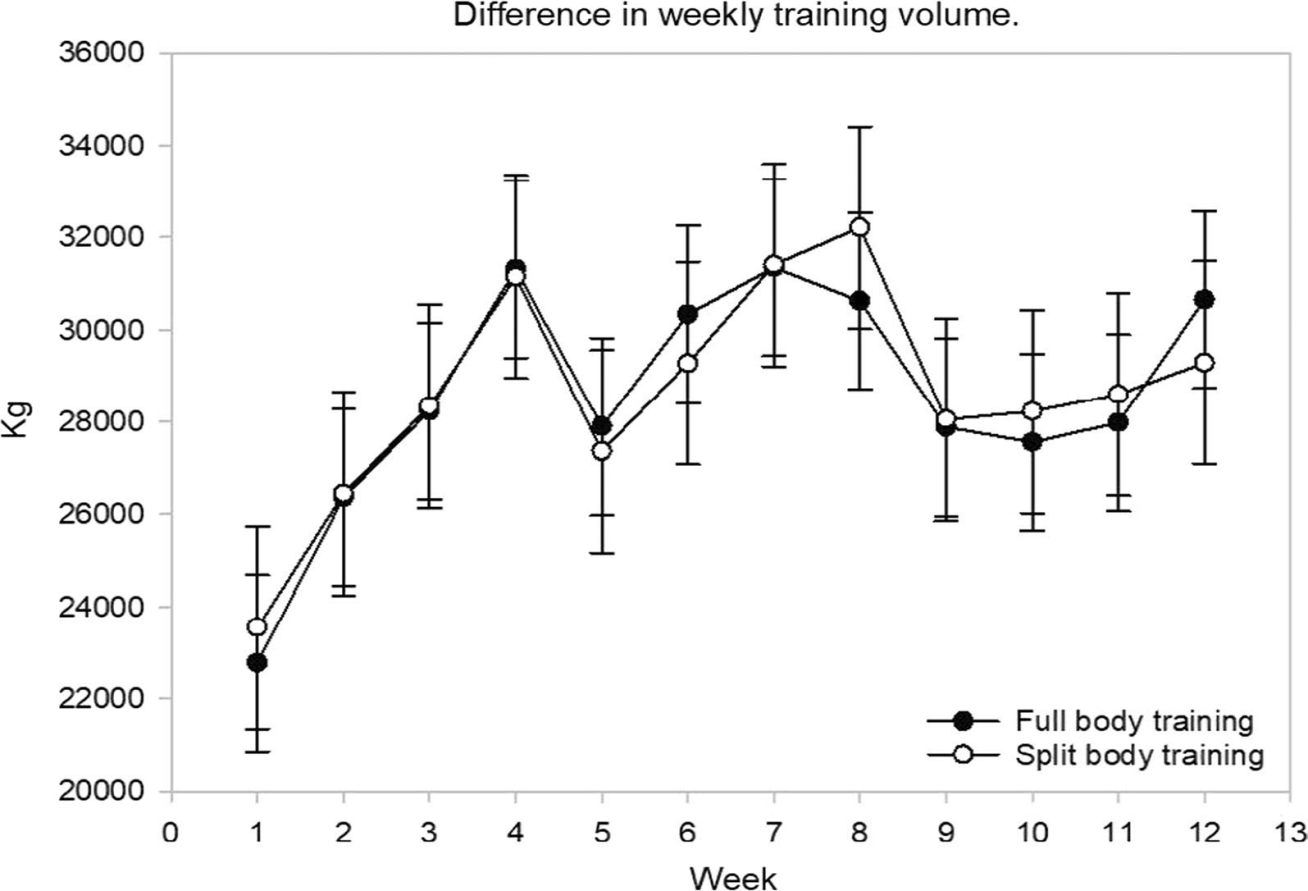
Weekly training volume throughout the intervention period. Results are presented as mean with 95% CI

Both groups increased their training volume throughout the intervention period, with no observed interaction or main effect. The mean difference in total kg lifted was 81 (− 2208, 2459) kg, *p* = 0.947). For FB, weekly training volume ranged from 22,771 (20,855, 24,687) kg to 31,348 (29,433, 33,265), with the lowest training volume noted during week 1 and the highest in week 7. For SPLIT, weekly training volume ranged from 23,543 (21,345, 25,741) kg to 32,215 (30,017, 34,412) kg, with the lowest training volume noted during week 1 and the highest in week 8. Figure 3 provides a complete illustration of weekly training volume throughout the intervention.

## Discussion

The aim of this study was to compare the effect of different training session frequencies (two versus four sessions per week) on muscle mass, muscle strength, jump height and power output, when each muscle group was trained an equal number of times (i.e. twice per week). Both groups achieved significant improvements in all study outcomes. However, no between-group differences were observed in any dependent variable, indicating that training frequency does not mediate muscular adaptations under the imposed conditions.

We hypothesized that the SPLIT group would increase muscle mass and maximal strength more than the FB group because shorter sessions could be performed at a higher intensity, resulting in increased total volume load over time. However, no differences were observed between groups in either total training volume, or when dividing the volume load into upper- and lower body components. The lack of difference in training volume load between the groups may explain our findings. The fact that the two groups performed a similar amount of work (i.e. total amount of kgs lifted) conceivably elicited a similar stimuli and consequently resulted in similar neuromuscular adaptions [28]. Hence, the findings of the current and previous studies indicate that session frequency is of minor consequence when employing a similar training volume [4, 6, 18, 19]. For example Ribeiro et al. [19] compared the effect of high training frequencies (six vs. four sessions) on muscular adaptations with a similar number of sessions per muscle group between groups in elite male bodybuilders. The results showed that when training muscle groups twice per week with an equal number of sets and reps, session frequency did not influence the adaptation in body composition or maximal strength in this population.

Not in line with our initial hypothesis, both groups showed similar improvements in explosive strength. Previous research has demonstrated a relationship between increases in 1-RM and improved jump height [23, 29–31]. Therefore, the lack of between-group differences in explosive parameters potentially could be explained by the similarities in muscle strength and muscle mass in the two groups, as power is developed on a foundation of strength. Furthermore, Hartman et al. [4] compared training once or twice a day with a similar training volume, in a cohort of nationally competetive weightlifters. Muscle strength, cross-sectional area, vertical jump peak power, hormone concentrations and EMG were measured before and after the 3 weeks intervention. There was no significant difference in jump performance between the groups, however, unlike the current study, Hartman et al. [4] did not observe any improvements in vertical jump peak power. Importantly, Hartman et al. [4] did not find an increase in maximal strength, which could explain the lack of improvement in jump height. Furthermore, the lack of improvement could be attributed to the fact that subjects were highly trained, the duration of training was very short (3 weeks), and the sample size was very small (five per group). To our knowledge, the present study is the first to investigate the effects of different session frequencies on explosive characteristics in the upper body.

The present study has some limitations that should be taken into consideration when interpreting the results. First, participants had little to no resistance training experience. It has previously been demonstrated that untrained individuals often respond well to all training programs regardless of training frequency [3, 32, 33]. Conceivably, training frequency may become more important when people gain experience in resistance training. Second, it is possible that a longer intervention with a higher total training volume could have resulted in different findings [34]. Usually, split-routines is a strategy to increase weekly training volume [5, 6], and it is conceivable that the volume we employed was insufficient to achieve desired benefits. Third, the session frequency in FB was 50% less than in SPLIT, and in Ribeiro et al. [19] the difference between the two groups was 33%. It is possible that greater contrasts (e.g. two vs. six sessions/week) are needed to elicit a difference. Fourth, although we instructed participants to maintain their usual and customary diets throughout the study period, we did not exert direct control over their nutritional intake. Although this raises the possibility that body composition data may have been confounded by dietary alterations, the relatively large sample size together with the fact that the groups were randomized, would seemingly have cancelled out any resulting differences between groups. Fifth, it can be argued that the inclusion of a non-training control group would have been beneficial. However, the effect of resistance training on measures of muscle strength and muscle mass is well-established and the purpose of this study was to compare two different training frequencies, and not the effectiveness of training per se. Finally, of the six participants who withdrew from the intervention, five were from the SPLIT group and four of them reported lack of time as the main reason. This raises the possibility that higher training frequency might not be ideal for an untrained population due to more days being occupied by training.

We conclude that distributing the exercises/sets over two full-body workouts or four upper/lower-body split-workouts promotes similar increases in maximal strength, muscle mass, upper-body power output and jump in non-resistance trained women when training each muscle group twice per week.

### Practical applications

The results from the present study indicate that two longer sessions are just as effective as four shorter sessions per week. Both training schedules have positive effects on maximal strength, muscle mass and explosive parameters. Therefore, untrained women can either choose to perform short sessions several days per week or train for a longer duration each session with fewer total training days per week based on personal preference. However, four participants in the high frequency group (SPLIT) versus none in the low frequency group (FB) dropped out due to motivational/time issues. Although this can be considered a relatively low percentage of the total participants in SPLIT, it nevertheless may be easier for young women to adhere to training using two longer sessions as compared to four shorter sessions.

## Data Availability

Data and materials can be sent on request to the corresponding author (helene.pedersen@hvl.no).

## Abbreviations

BMI: Body mass index
CI: Confidence interval
CMJ: Counter movement jump
FB: Full-body
ICC: Intraclass correlation coefficient
RM: Repetition maximum
SPLIT: Split-body
